# Influence of fat-free mass index on the survival of patients with head and neck cancer

**DOI:** 10.1007/s00405-022-07732-w

**Published:** 2022-11-27

**Authors:** Nina Lapornik, Brigita Avramovič Brumen, Gaber Plavc, Primož Strojan, Nada Rotovnik Kozjek

**Affiliations:** 1 Department of Clinical Nutrition, Institute of Oncology Ljubljana, Zaloška 2, 1000 Ljubljana, Slovenia; 2 Department of Radiation Oncology, Institute of Oncology Ljubljana, Zaloška 2, 1000 Ljubljana, Slovenia; 3 Faculty of Medicine, University of Ljubljana, Vrazov trg 2, 1000 Ljubljana, Slovenia; 4 Faculty of Health Sciences Izola, University of Primorska, Polje 42, 6310 Izola, Slovenia

**Keywords:** Fat-free mass index, Bioelectrical impedance analysis, Body mass index, Head and neck cancer, Overall survival

## Abstract

**Purpose:**

To determine whether muscle mass, defined by fat-free mass index (FFMI) measured with bioelectrical impedance analysis (BIA), is predictive of survival of head and neck squamous cell carcinoma (HNSCC) patients.

**Methods:**

HNSCC patients treated between 2014 and 2018 at the Department for Nutrition of the Institute of Oncology Ljubljana were reviewed. The FFMI values from the pretreatment BIA measurements and pretreatment body mass index (BMI) were used to categorize patients into groups with low and normal muscle mass and BMI using the Global Leadership Initiative on malnutrition (GLIM) recommended cutoff values. The impact of FFMI on disease-free survival (DFS) and overall survival (OS) was determined.

**Results:**

Of the 71 included patients, 31 (43.7%) had normal FFMI, and 40 (56.3%) had low FFMI, whereas 44 (62%) and 27 (38%) of the patients had normal and low BMI, respectively. Between FFMI and BMI values, a significant correlation was found (*R*_P_ = 0.75, *p* < 0.001). Univariate regression analysis showed that FFMI (as a continuous variable) was of prognostic significance for OS (*p* = 0.039), which was confirmed by multivariate regression analysis (*p* = 0.029). The model where BMI replaced FFMI negated the prognostic value of BMI (as a continuous variable). Neither FFMI nor BMI was found to be a predictor of DFS on univariate or multivariate analysis.

**Conclusions:**

In the present group of HNSCC patients, low FFMI adversely influenced OS, emphasizing the importance of using body composition measurement over BMI alone for pretreatment nutritional evaluation of these patients.

## Introduction

Head and neck squamous cell carcinoma (HNSCC) patients are often nutritionally compromised due to lifestyle, location of the tumor growth, and the effects of treatment on food intake [1]. HNSCC patients have the second highest prevalence of malnutrition, with pretreatment severe weight loss ranging between 19% and 57% [2–4].

Malnutrition leads to altered body composition with depletion of fat mass and lean body mass, resulting in reduced physical and mental functioning and poorer clinical outcome [5]. Approximately 70% of weight loss in cancer patients is thought to be due to loss of lean body mass [6–8]. Reduction of skeletal muscle mass is a good indicator of lean body mass loss and one of the established diagnostic criteria for assessing nutritional status [5, 9]. It leads to an increased risk of rehospitalizations, falls, fractures, loss of independence and death in hospitalized patients [10, 11].

Several studies in patients with HNSCC found an association between computed tomography (CT) determined decreased muscle mass and worse survival [12–15]. Although CT scans could be an important tool for assessing muscle mass [16–18], they are rarely used in clinical routine for this purpose [19]. According to the Global Leadership Initiative on Malnutrition (GLIM) criteria from 2019, reduced muscle mass is one out of three possible phenotypic criteria for diagnosing malnutrition in cancer patients and can be determined by fat-free mass index (FFMI) measurement using bioelectrical impedance analysis (BIA) [20].

Currently, BIA is a widely available, simple, non-invasive, and inexpensive method, routinely used in clinical settings [21]. Measuring the impedance of body tissues to the flow of electric current at a fixed frequency or range of frequencies determines the electrically conductive properties of the body and predicts body composition [22]. The principle of BIA is that lean tissue, consisting of water and electrolytes, is a good electrical conductor; on the contrary, fat is a poor electrical conductor as it does not contain water. Fat-free mass (FFM) assessed by BIA using special regression equations calibrated against the direct measurement of FFM can be and is used for FFMI calculation [23].

Under standard conditions, BIA measurements showed good correlation with the assessment of muscle mass by dual-energy X-ray absorptiometry (DEXA) [24, 25], magnetic resonance imaging (MRI) [26], and CT [27]. In HNSCC patients, body composition as determined with BIA was found to correlate strongly with CT-based estimates, although HNSCC patients represent a challenging population given wide fluctuations in their hydration status [19]. However, information on the impact of BIA-derived FFMI on the disease-free survival (DFS) and overall survival (OS) of HNSCC patients is limited in the literature [28]. The aim of the present study was to determine whether the FFMI determined by BIA can be used as a prognosticator for DFS and OS in this challenging group of patients.

## Subjects and methods

### Patient eligibility

The study retrospectively included patients with HNSCC treated with curative intent between 2014 and 2018 who had pretreatment BIA (Bodystat^®^ Quadscan 4000 (Douglas, GB)) evaluation of their nutritional status at the Department for Clinical Nutrition of Institute of Oncology Ljubljana, Slovenia. All tumors were histologically confirmed and without systemic metastases located in the oral cavity, oropharynx, hypopharynx, or larynx. Patients were treated with definitive or postoperative (chemo)radiotherapy (RT) and had to complete their treatment as planned. Linac-based intensity-modulated radiotherapy and concurrent weekly cisplatin (40 mg/m^2^ IV, in patients at high risk for in-field recurrence) were employed as indicated by the Multidisciplinary Head and Neck Tumor Board. Exclusion criteria were prior treatment in the head and neck area and any synchronous cancer except basal cell carcinoma of the skin.

### Study design

Demographic data and tumor-, treatment- and survival-related information were extracted from the clinical records of the patients. The tumors were staged using the criteria of the International Union Against Cancer (UICC) TNM staging system, 7th edition [29]. The p16 and/or human papillomavirus (HPV) status in patients with oropharyngeal cancer was determined by immunohistochemistry and/or in situ hybridization studies. Patients who had stopped smoking more than 2 years prior to diagnosis were considered ex-smokers. Comorbidities of patients at the time of HNSCC diagnosis were assessed using Charlson comorbidity Index, where the index cancer was not considered comorbidity [30].

FFMI was determined during the first consultation with a clinical dietitian using BIA, which was performed with the BodyStat BIA device (Douglas, GB) according to the standards of the National Health Institute, as previously described [31, 32]. To differentiate between normal and reduced FFMI values, cutoff points determined by the GLIM criteria for malnutrition were employed: for men, < 17 kg/m^2^ and for women < 15 kg/m^2^ [20]. Body mass index (BMI) values were also calculated and categorized according to the GLIM criteria (low BMI, < 70 years: < 20 kg/m^2^; > 70 years: < 22 kg/m^2^) [20].

### Statistical methods

The study was conducted according to the guidelines of the Declaration of Helsinki, and the study protocol was approved by the Committee for Medical Ethics and the Protocol Review Board of the Institute of Oncology Ljubljana (ERIDEK-0044/2021, 21.5.2021).

Statistical analysis was performed using R Studio, version 1.4.1106 (R-3.6.3). Categorical variables are presented as frequencies, and for continuous variables, arithmetic mean, standard deviation and range were calculated. The association of FFMI with categorical and continuous variables was tested with the chi-squared test (or Fisher’s exact test if the number of subjects in any of the cells was < 5) and the t test, respectively. The parametric correlation test (Pearson) was used to measure a linear dependence between the FFMI and BMI values in individual patients. The aims of the survival analysis were DFS (locoregional failure, distant metastasis, or death from any cause considered as an event) and OS (death from any cause considered as an event), which were defined as the time between the date of histological verification of the tumor and event or close-out date. The probability of DFS and OS was assessed using the Kaplan‒Meier method, and the log-rank test was used for curve comparison. The influence of FFMI and other variables on the OS of patients was tested with the univariate Cox regression model, where the FFMI and BMI were analyzed as continuous and categorical variables. Because several different covariates can potentially affect patient prognosis, a multivariate Cox regression model was used to examine the effect of different variables. In this model, the effect of FFMI and BMI on DFS and OS was examined separately, considering other variables that showed an impact (*p* < 0.1) on patient survival in univariate analysis. Thus, several models with different sets of variables were used in the multivariate analysis of DFS and OS. The performance of different models was compared by the corrected Akaike information criterion (cAIC), a tool for assessing the quality of various statistical models relative to each other and for the selection of the best model [33]. A *p* value of ≤ 0.05 was considered statistically significant.

## Results

### Study population

Out of 569 patients, 71 patients fulfilled the inclusion criteria. The overall mean FFMI value of all patients was 16.4 kg/m^2^ (SD ± 2.6, range 10.7–4.1), in women 14.0 kg/m^2^ (SD ± 2.2, range 10.7–17.0) and in men 17.0 kg/m^2^ (SD ± 2.4, range 11.8–24.1). Thirty-one (43.7%) patients had normal FFMI, and 40 (56.3%) patients had low FFMI. Considering BMI, 44 (62%) patients were classified into the group with a normal BMI and 27 (38%) into the group with a reduced BMI. A significant correlation was found between FFMI and BMI values measured in individual patients (*R*_P_ = 0.75, 95% confidence interval [CI] 0.63–0.84, *p* < 0.001). The demographic, clinical and nutritional characteristics of the study group are shown in Table 1. Smokers and ex-smokers were more likely to have low FFMI (*p* = 0.003), which was also associated with low BMI (*p* < 0.001).

**Table 1:** Demographic and clinical characteristics of patients grouped by fat free mass index (low: men, < 17 kg/m^2^; women, < 15 kg/m^2^)

Characteristic	All (*n* = 71)	Normal FFMI (*n* = 31; 43.7%)	Low FFMI (*n* = 40; 56.3%)	*p* value
Age				
Mean age (± SD)	61 (12.1)	60.89 (13.60)	61.10 (11.00)	0.945
Sex				
Men	56 (78.9%)	25 (80.6%)	31 (77.5%)	0.748
Women	15 (21.1%)	6 (19.4%)	9 (22.5%)	
Performance status (WHO)				
0–1	55 (77.5%)	23 (74.2%)	32 (80.0%)	0.561
2	16 (22.5%)	8 (25.8%)	8 (20.0%)	
BMI				
Mean BMI (± SD)	22.25 (4.12)	25.68 (3.39)	19.59 (2.24)	**< 0.001**
Low BMI	27 (38%)	0 (0%)	27 (67.5%)	**< 0.001**
Normal BMI	44 (62%)	31 (100%)	13 (32.5%)	
Smoking status (*n* = 63)				
Non-smokers and ex-smokers	22 (31%)	15 (55.6%)	7 (19.4%)	**0.003**
Smokers	41 (57.7%)	12 (44.4%)	29 (80.6%)	
Unknown	8 (11.3%)			
Comorbidities (CCI)				
0	48 (67.6%)	18 (58.1%)	30 (75.0%)	0.130
1–3	23 (32.4%)	13 (41.9%)	10 (25.0%)	
1	12 (16.9%)			
2	6 (8.5%)			
3	5 (7.0%)			
Primary tumor location				
Oropharynx	32 (45.1%)	12 (38.7%)	20 (50.0%)	0.374
HPV +	8 (25%)			
HPV−	24 (75%)			
Hypopharynx and larynx	27 (38.1%)	13 (41.9%)	14 (35.0%)	
Oral cavity	12 (16.9%)	6 (19.4%)	6 (15.0%)	
Overall stage				
I–III	13 (18.3%)	8 (25.8%)	5 (12.5%)	0.151
IV	58 (81.7%)	23 (74.2%)	35 (87.5%)	
Surgical resection				
Yes	32 (45.1%)	18 (58.1%)	14 (35.0%)	0.053
R0	27 (84.4%)			
R1	2 (6.2%)			
R2	3 (9.4%)			
No	39 (54.9%)	13 (41.9%)	26 (65.0%)	
Addition of ChT				
RT	32 (45.1%)	17 (54.8%)	15 (37.5%)	0.069
ChRT	36 (50.7%)	14 (45.2%)	22 (55.0%)	
Induction ChT (→ RT/ChRT)	3 (4.2%)	0 (0%)	3 (7.5%)	

*FFMI* fat free mass index, *BMI* body mass index, *SD* standard deviation, *WHO* World Health Organization, *HPV* Human papillomavirus, *ChRT* Chemoradiotherapy, *RT* radiotherapy, *ChT* chemotherapy, *BIA* bioelectrical impedance analysis

*p* ≤ 0.05 statistically significant are in bold

On the close-out date, 53 (74.6%) of the patients were dead, either due to disease progression (25, 35%) or other causes (28, 39.4%). The mean time to malignant disease progression/recurrence or death was 1.5 years (range 0–5.6). Surviving patients were followed-up between 2.6 and 7.4 years (mean 4.4).

**Fig. 1:**
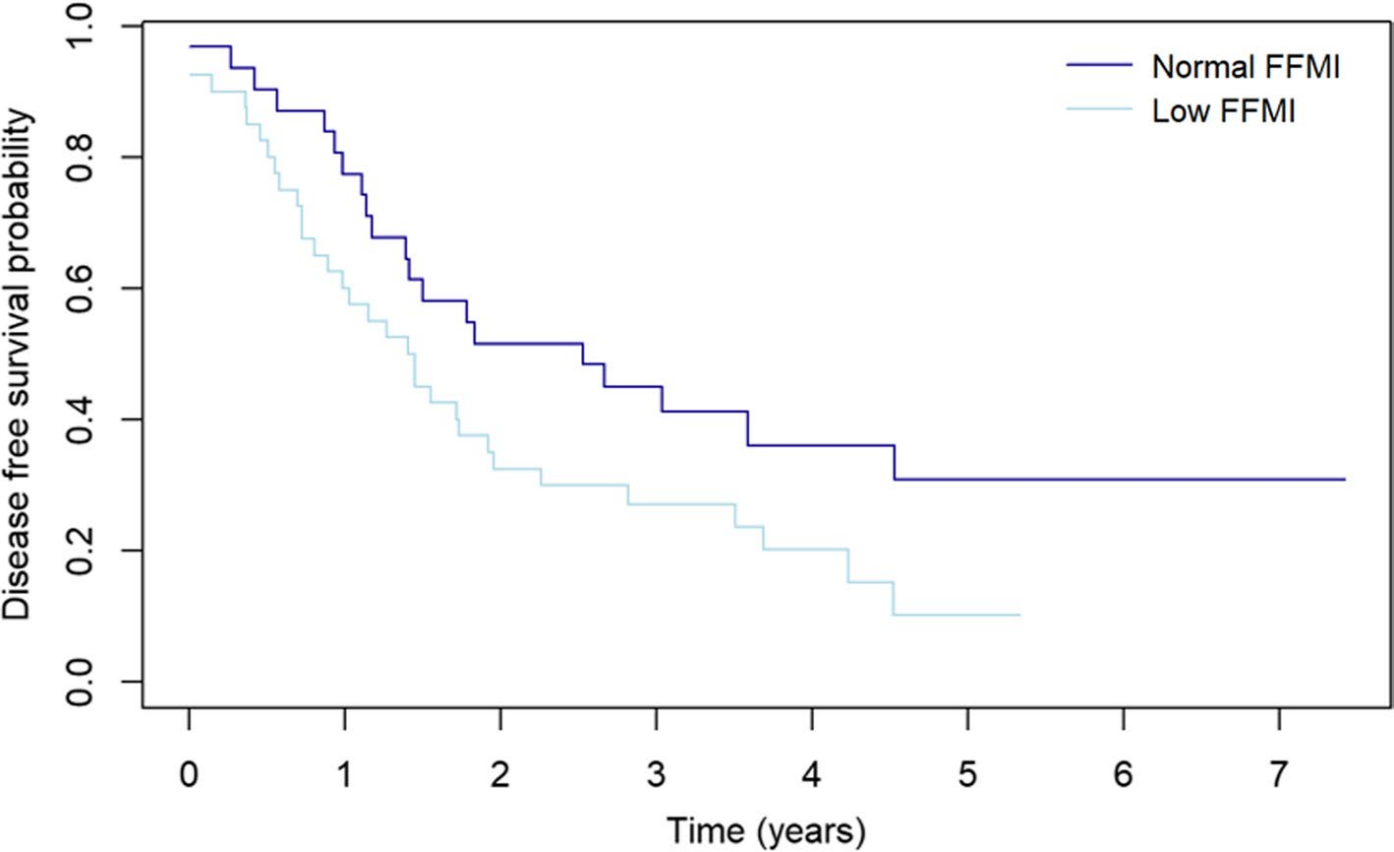
Disease-free survival of patients with low and normal fat-free mass index (FFMI) as determined by bioelectrical impedance analysis (BIA) (*p* = 0.06)

**Fig. 2:**
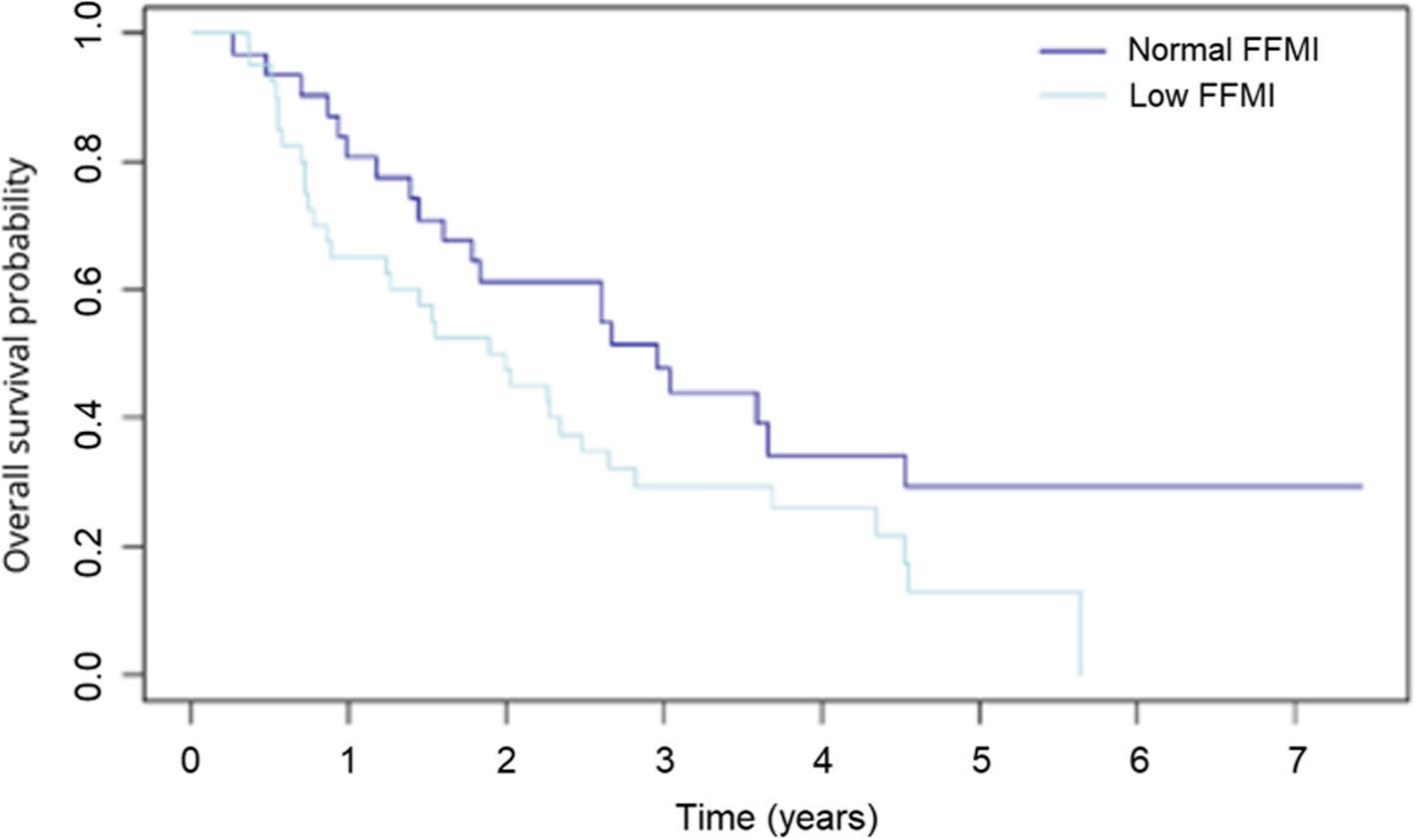
Overall survival of patients with low and normal fat-free mass index (FFMI) as determined by bioelectrical impedance analysis (BIA) (*p* = 0.06)

The DFS rates at 3 years of patients with low and normal FFMI was 27.0% (95% CI 0.16–0.45) and 44.9% (95% CI 0.30–0.67), respectively (*p* = 0.06, Fig. 1) and the OS rates 29.4% (95% CI 0.18–0.48) and 47.7% (95% CI 0.33–0.69), respectively (*p* = 0.06, Fig. 2). DFS was 36.2% (95% CI 0.24–0.54) in patients with normal BMI and 32.4% (95% CI 0.19–0.57) in those with low BMI (*p* = 0.80). The corresponding OS rates were 40.5% (95% CI 0.28–0.58) and 32.1% (95% CI 0.18–0.56), respectively (*p* = 0.60).

In the univariate Cox regression model for DFS, only treatment type (surgical vs. non-surgical, *p* = 0.038) had a statistically significant effect (Table 2). In three different multivariate analysis models, only PS consistently showed statistical significance on DFS: neither FFMI (as a continuous or binary variable), nor BMI (continuous) was retained in the final model (Table 2).

**Table 2:** Univariate and multivariate Cox regression analyses of disease-free survival (*n* = 71)

Variable	Univariate analysis		Multivariate analysis 1		Multivariate analysis 2		Multivariate analysis 3	
	HR (95% CI)	*p* value	HR (95% CI)	*p* value	HR (95% CI)	*p* value	HR (95% CI)	*p* value
FFMI (normal vs. low)	1.72 (0.98–3.01)	0.058			1.96 (0.75–5.11)	0.171		
FFMI (continuous)	0.90 (0.81–1.00)	0.060	0.96 (0.78–1.18)	0.691				
BMI (normal vs. low)	1.09 (0.62–1.89)	0.771						
BMI (continuous)	0.96 (0.89–1.02)	0.185					0.92 (0.81–1.04)	0.174
Sex (men vs. women)	1.19 (0.63–2.27)	0.593						
Age (continuous)	1.01 (0.99–1.03)	0.592						
Comorbidities (0 vs. 1–3)	1.46 (0.82–2.59)	0.200						
Tumor location (OP/OC vs. HP/LX)	1.02 (0.59–1.78)	0.939						
Overall stage (I–III vs. IV)	1.40 (0.66–2.98)	0.378						
Treatment type (surgical vs. non-surgical)	1.79 (1.03–3.11)	**0.038**	0.98 (0.34–2.84)	0.960	0.79 (0.27–2.32)	0.674	1.12 (0.41–3.07)	0.833
Performance status, WHO (0–1 vs. 2)	1.71 (0.93–3.16)	0.086	4.75 (1.51–14.97)	**0.008**	4.50 (1.45–13.99)	**0.009**	5.12 (1.63–16.08)	**0.005**
Smoking status (non-/ex-smokers vs. smokers)	1.04 (0.57–1.90)	0.89						
p16/HPV status (negative vs. positive),* n* = 32*	0.35 (0.18–1.05)	0.06	0.44 (0.13–1.50)	0.190	0.39 (0.11–1.38)	0.136	0.45 (0.13–1.53)	0.201

*FFMI* fat free mass index, *BMI* body mass index, *OP* oropharynx, *OC* oral cavity, *HP* hypopharynx, *LX* larynx, *WHO* World Health Organization, *n* number of patients, *HR* hazard ratio, *CI* confidence interval

*p* ≤ 0.05 statistically significant are in bold

*Only patients with oropharyngeal primary tumors

In the univariate Cox regression model, World Health Organization (WHO) performance status (PS) (0–1 vs. 2, *p* = 0.016) and FFMI (as a continuous variable, *p* = 0.039) had a statistically significant effect on OS (Table 2). In the first multivariable analysis model, which included FFMI as a continuous variable, both variables remained statistically significant. In the second model with BMI (as a continuous variable), only PS and treatment type showed statistical significance (Table 3). For both multivariate analysis models, the cAIC was obtained to reveal the model with the lowest cAIC value (Table 4). The model that included FFMI was shown to be more accurate and informative in terms of OS prediction than the model with BMI.

**Table 3:** Univariate and multivariate Cox regression analyses of overall survival (*n* = 71)

Variable	Univariate analysis		Multivariate analysis 1		Multivariate analysis 2	
	Hazard ratio (95% CI)	*p* value	Hazard ratio (95% CI)	*p* value	Hazard ratio (95% CI)	*p* value
FFMI (normal vs. low)	1.71 (0.98–2.99)	0.06				
FFMI (continuous)	0.89 (0.81–0.99)	**0.039**	0.88 (0.79–0.99)	**0.029**		
BMI (normal vs. low)	1.17 (0.67–2.05)	0.582				
BMI (continuous)	0.95 (0.89–1.02)	0.15			0.93 (0.86–1.00)	0.061
Sex (men vs. women)	1.15 (0.60–2.18)	0.677				
Age (continuous)	1.01 (0.98–1.03)	0.525				
Tumor location (OP/OC vs. HP/LX)	1.00 (0.57–1.74)	0.991				
Overall stages (I–III vs. IV)	1.63 (0.77–3.47)	0.203				
Treatment type (surgical vs. non-surgical)	1.65 (0.96–2.86)	0.072	1.64 (0.92–2.91)	0.091	1.92 (1.08–3.39)	**0.025**
Performance status, WHO (0–1 vs. 2)	2.14 (1.15–3.98)	**0.016**	2.85 (1.48–5.47)	**0.002**	2.90 (1.49–5.64)	**0.002**
Smoking status (non-/ex-smokers vs. smokers)	0.92 (0.50–1.68)	0.788				
P16/HPV status (negative vs. positive), n = 32*	0.43 (0.15–1.27)	0.127				

*FFMI* fat free mass index, *BMI* body mass index, *OP* oropharynx, *OC* oral cavity, *HP* hypopharynx, *LX* larynx, *WHO* World Health Organization, *n* number of patients, *CI* confidence interval

*p* ≤ 0.05 statistically significant are in bold

*Only patients with oropharyngeal primary tumors

**Table 4:** Prognostic performance of multivariate analysis models

Model	K	AICc
Overall survival		
MVA model with FFMI as a continuous variable	3	376.42
MVA model with BMI as a continuous variable	3	377.46
Disease-free survival		
MVA model with FFMI as a continuous variable	4	134.08
MVA model with FFMI as a categorical variable	4	134.08
MVA model with BMI as a continuous variable	4	135.89

*MVA* multivariable analysis, *FFMI* fat free mass index, *BMI* body mass index, *K* number of parameters in the model, *cAIC* corrected Akaike information criterion

## Discussion

The present study confirms that body composition as measured by BIA, but not BMI, is an independent prognostic factor for predicting OS in HNSCC patients, in addition to their PS. This speaks in favor of BIA as a more practical bedside procedure that, e.g., CT, is also noninvasive, reproducible and inexpensive [21, 34]. Although BIA may result in incorrect assessment of muscle mass with FFMI in poorly hydrated patients [34], a good correlation was generally reported between BIA and CT measurements of skeletal muscle mass [19].

The GLIM consensus for malnutrition recognized 3 phenotypic criteria for the diagnosis of malnutrition, i.e., weight loss (in %), decrease in muscle mass and BMI [20]. Before the start of treatment, 38% of our patients had a low BMI. After categorizing patients according to GLIM criteria, 56.3% of the patients had reduced pretreatment FFMI. This almost 20% difference in the share of malnourished patients further supports the importance of the use of several criteria to determine malnutrition [20]. In studies that used either CT scans or BIA for the determination of muscle mass, the prevalence of patients with low muscle mass differs significantly (20.5–54.5%) [15, 35], reflecting the characteristics of the studied population. In our case, only HNSCC patients who were directed to our department before oncological treatment due to clinically identifiable and already existing or threatened malnutrition were included in the study. This could be the reason for the higher proportion of patients with low FFMI than in some other studies [26, 35, 36].

We found no correlation between FFMI and age, which is contrary to the general premise that muscle mass decreases with age [15, 37]. Furthermore, the FFMI of our patients also did not correlate with sex, PS, primary tumor location, overall disease stage, or type of treatment (definitive or postoperative (chemo)radiotherapy). This also contradicts the findings of some other authors, i.e., the relationship between low muscle mass and female sex [15, 38, 39] or higher overall disease stage [14, 38]. However, we observed an association between reduction of muscle mass and smoking, as did Bril et al. [38] but not also Wendrich et al. [15] and Huiskamp et al. [40], possibly reflecting the problem of the reliability of data obtained from patients. In addition, reduced muscle mass correlated with a lower mean BMI in our patients, which was also previously described [15, 38].

The 3-year DFS and OS in our group of HNSCC patients were only 34.9% and 37.5%, respectively, probably because of a selection bias by including mainly already nutritionally compromised patients (94.4%) in the advanced stage of the disease (IVA-B, 81.7%) with at least one comorbidity (32.4%). This probably masked the differences in survival between individual categories of patients (e.g., regarding the origin of the tumor, the stage of the disease, the type of treatment). An additional, albeit related, reason was the high prevalence of low muscle mass (56.3%) in our patients, which turned out to be an independent adverse prognostic factor for OS (but not also for DFS) in multivariate analysis. Several other studies demonstrated a negative prognostic impact of low muscle mass on the survival of HNSCC patients [14, 19, 39]. Moreover, despite the statistically significant correlation between FFMI and BMI values measured in individual patients, only FFMI proved to be of significance for predicting OS in multivariate analysis. Although the association between reduced muscle mass and weight reduction (and thus lower BMI) is to be expected, it should be noted that BMI alone is not a good predictor of lower muscle mass and altered body composition [41]. Moreover, one should be aware that weight loss is not necessarily present in sarcopenia and, on the other hand, muscle mass may also be reduced in individuals with sarcopenic obesity [42]. In contrast to the OS analysis, neither FFMI nor BMI was found to be an independent predictor of DFS.

Limitations of this study relate primarily to its retrospective nature and its inherent shortcomings (selection bias, sometimes deficient data that are questionably reliable). Furthermore, inclusion criteria that further curtailed the available set of patients and resulted in a relatively small sample size added to the selection bias and resulted in a problem with the relativity of small series statistics. For a reliable determination of the relationship between FFMI and OS, all patients with HNSCC treated over a selected period should have been included. However, due to the lack of research in HNSCC using BIA-determined FFMI, the presented results could be a valuable source of scientific data to the existing knowledge.

To conclude, in the present study, we retrospectively demonstrated that BIA determined low FFMI as a measure of body muscle mass, but BMI also did not appear to be a negative prognostic factor for OS in HNSCC patients. This emphasizes the importance of using body composition measurements, such as FFMI, over BMI alone in these patients for prognostic evaluation. Although our findings are consistent with the general opinion of the experts that low muscle mass is prognostic for negative oncological outcomes, further studies with prospective recruitment of all HNSCC patients are needed for confirmation.

## Data Availability

The datasets analyzed during the current study are available from the corresponding author on reasonable request.

## References

[1] Almada-Correia I, Neves PM, Mäkitie A, Ravasco P (2019). Body composition evaluation in head and neck cancer patients: a review. Front Oncol.

[2] Ferrão B, Neves PM, Santos T, Capelas ML, Mäkitie A, Ravasco P (2020). Body composition changes in patients with head and neck cancer under active treatment: a scoping review. Support Care Cancer.

[3] Jager-Wittenaar H, Dijkstra PU, Vissink A, van der Laan BFAM, van Oort RP, Roodenburg JLN (2007). Critical weight loss in head and neck cancer-prevalence and risk factors at diagnosis: an explorative study. Support Care Cancer.

[4] van Bokhorst-de van der Schueren MA, van Leeuwen PA, Sauerwein HP, Kuik DJ, Snow GB, Quak JJ (1997) Assessment of malnutrition parameters in head and neck cancer and their relation to postoperative complications. Head Neck 19:419–425. 10.1002/(sici)1097-0347(199708)19:5<419::aid-hed9>3.0.co;2-2 10.1002/(sici)1097-0347(199708)19:5<419::aid-hed9>3.0.co;2-2 9243270

[5] Cederholm T, Barazzoni R, Austin P, Ballmer P, Biolo G, Bischoff SC (2017). ESPEN guidelines on definitions and terminology of clinical nutrition. Clin Nutr.

[6] Jackson W, Alexander N, Schipper M, Fig L, Feng F, Jolly S (2014). Characterization of changes in total body composition for patients with head and neck cancer undergoing chemoradiotherapy using dual-energy x-ray absorptiometry. Head Neck.

[7] Solís-Martínez O, Plasa-Carvalho V, Phillips-Sixtos G, Trujillo-Cabrera Y, Hernández-Cuellar A, Queipo-García GE, Meaney-Mendiolea E, Ceballos-Reyes GM, Fuchs-Tarlovsky V (2018). Effect of eicosapentaenoic acid on body composition and inflammation markers in patients with head and neck squamous cell cancer from a public hospital in Mexico. Nutr Cancer.

[8] Lønbro S, Dalgas U, Primdahl H, Johansen J, Nielsen JL, Aagaard P, Hermann AP, Overgaard J, Overgaard K (2013). Progressive resistance training rebuilds lean body mass in head and neck cancer patients after radiotherapy: results from the randomized DAHANCA 25B trial. Radiother Oncol.

[9] Landi F, Camprubi-Robles M, Bear DE, Cederholm T, Malafarina V, Welch AA, Cruz-Jentoft AJ (2019). Muscle loss: the new malnutrition challenge in clinical practice. Clin Nutr.

[10] Prado CM, Purcell SA, Alish C, Pereira SL, Deutz NE, Heyland DK, Goodpaster BH, Tappenden KA, Heymsfield SB (2018). Implications of low muscle mass across the continuum of care: a narrative review. Ann Med.

[11] Gariballa S, Alessa A (2017). Impact of poor muscle strength on clinical and service outcomes of older people during both acute illness and after recovery. BMC Geriatr.

[12] Nishikawa D, Hanai N, Suzuki H, Koide Y, Beppu S, Hasegawa Y (2018). The impact of skeletal muscle depletion on head and neck squamous cell carcinoma. ORL.

[13] Thureau S, Lebret L, Lequesne J, Cabourg M, Dandoy S, Gouley C, Lefebvre L, Mallet R, Mihailescu SD, Moldovan C, Rigal O, Veresezan O, Modzewelski R, Clatot F (2021). Prospective evaluation of sarcopenia in head and neck cancer patients treated with radiotherapy or radiochemotherapy. Cancers.

[14] Rijn-Dekker MI, Bosch L, Hoek JGM, Bijl HP, Aken ESM, Hoorn A, Oosting SF, Halmos GB, Witjes MJH, van der Laan HP, Langendijk JA, Steenbakkers RJHM (2020). Impact of sarcopenia on survival and late toxicity in head and neck cancer patients treated with radiotherapy. Radiother Oncol.

[15] Wendrich AW, Swartz JE, Bril SI, Wegner I, de Graeff A, Smid EJ, de Bree R, Pothen AJ (2017). Low skeletal muscle mass is a predictive factor for chemotherapy dose-limiting toxicity in patients with locally advanced head and neck cancer. Oral Oncol.

[16] Shen W, Punyanitya M, Wang Z, Gallagher D, St-Onge M-P, Albu J, Heymsfield SB (1985). Heshka S (2004) Total body skeletal muscle and adipose tissue volumes: estimation from a single abdominal cross-sectional image. J Appl Physiol.

[17] Mourtzakis M, Prado CMM, Lieffers JR, Reiman T, McCargar LJ, Baracos VE (2008). A practical and precise approach to quantification of body composition in cancer patients using computed tomography images acquired during routine care. Appl Physiol Nutr Metab.

[18] Cesari M, Vellas B (2012). Sarcopenia: a novel clinical condition or still a matter for research?. J Am Med Dir Assoc.

[19] Grossberg AJ, Rock CD, Edwards J, Mohamed ASR, Ruzensky D, Currie A, Rosemond P, Phan J, Gunn GB, Frank SJ, Morrison WH, Garden AS, Fuller CD, Rosenthal DI (2021). Bioelectrical impedance analysis as a quantitative measure of sarcopenia in head and neck cancer patients treated with radiotherapy. Radiother Oncol.

[20] Cederholm T, Gl J, Correia MITD, Gonzalez MC, Fukushima R, Higashiguchi T (2019). GLIM criteria for the diagnosis of malnutrition—a consensus report from the global clinical nutrition community. J Cachexia Sarcopenia Muscle.

[21] Deutz NEP, Ashurst I, Ballesteros MD, Bear DE, Cruz-Jentoft AJ, Genton L, Landi F, Laviano A, Norman K, Prado CM (2019). The underappreciated role of low muscle mass in the management of malnutrition. J Am Med Dir Assoc.

[22] Kuriyan R (2018). Body composition techniques. Indian J Med Res.

[23] Gonzalez MC, Pastore C, Orlandi S, Heymsfield S (2014). Obesity paradox in cancer: new insights provided by body composition. Am J Clin Nutr.

[24] Leahy S, O’Neill C, Sohun R, Jakeman P (2012). A comparison of dual energy X-ray absorptiometry and bioelectrical impedance analysis to measure total and segmental body composition in healthy young adults. Eur J Appl Physiol.

[25] Kim M, Shinkai S, Murayama H, Mori S (2015). Comparison of segmental multifrequency bioelectrical impedance analysis with dual-energy X-ray absorptiometry for the assessment of body composition in a community-dwelling older population. Geriatr Gerontol Int.

[26] Janssen I, Heymsfield SB, Baumgartner RN (1985). Ross R (2000) Estimation of skeletal muscle mass by bioelectrical impedance analysis. J Appl Physiol.

[27] Aleixo GFP, Shachar SS, Nyrop KA, Muss HB, Battaglini CL, Williams GR (2020). Bioelectrical impedance analysis for the assessment of sarcopenia in patients with cancer: a systematic review. Oncologist.

[28] Willemsen ACH, Hoeben A, Lalisang RI, Van Helvoort A, Wesseling FWR, Hoebers F, Baijens LWJ, Schols AMWJ (2020). Disease-induced and treatment-induced alterations in body composition in locally advanced head and neck squamous cell carcinoma. J Cachexia Sarcopenia Muscle.

[29] Sobin LH, Gospodarowicz M, Wittekind C (2003). International Union Against Cancer (UICC) (2010) TNM Classification of Malignant Tumours.

[30] Charlson ME, Pompei P, Ales KL, MacKenzie R (1987). A new method of classifying prognostic comorbidity in longitudinal studies: development and validation. J Chron Dis.

[31] Gosak M, Gradišar K, RotovnikKozjek RN, Strojan P (2020). Psychological distress and nutritional status in head and neck cancer patients: a pilot study. Eur Arch Otorhinolaryngol.

[32] Stegel P, Kozjek NR, Brumen BA, Strojan P (2016). Bioelectrical impedance phase angle as indicator and predictor of cachexia in head and neck cancer patients treated with (chemo)radiotherapy. Eur J Clin Nutr.

[33] Cavanaugh JE, Neath AA (2019). The Akaike information criterion: Background, derivation, properties, application, interpretation, and refinements. WIREs Comput Stat.

[34] Cruz-Jentoft AJ, Bahat G, Bauer J, Boirie Y, Bruyère O, Cederholm T (2019). Writing Group for the European Working Group on Sarcopenia in Older People 2 (EWGSOP2), and the Extended Group for EWGSOP2. Sarcopenia: revised European consensus on definition and diagnosis. Age Ageing.

[35] Cereda E, Pedrazzoli P, Lobascio F, Masi S, Crotti S, Klersy C, Turri A, Stobäus N, Tank M, Franz K, Cutti S, Giaquinto E, Filippi AR, Norman K, Caccialanza R (2021). The prognostic impact of BIA-derived fat-free mass index in patients with cancer. Clin Nutr.

[36] Lundberg M, Nikander P, Tuomainen K, Orell-Kotikangas H, Mäkitie A (2017). Bioelectrical impedance analysis of head and neck cancer patients at presentation. Acta Oto-Laryngol.

[37] Janssen I, Heymsfield SB, Wang ZM, Ross R (2000). Skeletal muscle mass and distribution in 468 men and women aged 18–88 yr. J Appl Physiol (1985).

[38] Bril SI, Pezier TF, Tijink BM, Janssen LM, Braunius WW, de Bree R (2019). Preoperative low skeletal muscle mass as a risk factor for pharyngocutaneous fistula and decreased overall survival in patients undergoing total laryngectomy. Head Neck.

[39] Chargi N, Wegner I, Markazi N, Smid E, de Jong P, Devriese L, de Bree R (2021). Patterns, predictors, and prognostic value of skeletal muscle mass loss in patients with locally advanced head and neck cancer undergoing cisplatin-based chemoradiotherapy. J Clin Med.

[40] Huiskamp LFJ, Chargi N, Devriese LA, de Jong PA, de Bree R (2020). The predictive and prognostic value of low skeletal muscle mass for dose-limiting toxicity and survival in head and neck cancer patients receiving concomitant cetuximab and radiotherapy. Eur Arch Otorhinolaryngol.

[41] Gonzalez MC, Correia MITD, Heymsfield SB (2017). A requiem for BMI in the clinical setting. Curr Opin Clin Nutr Metab Care.

[42] Donini LM, Busetto L, Bischoff SC, Cederholm T, Ballesteros-Pomar MD, Batsis J (2022). Definition and diagnostic criteria for sarcopenic obesity: ESPEN and EASO consensus statement. Obes Facts.

